# Survey of Sand Flies (Diptera: Psychodidae) in an Environmentally Protected Area in Brazil

**DOI:** 10.1371/journal.pone.0134845

**Published:** 2015-08-12

**Authors:** Lara Saraiva, Alanna Silva Reis, Jeronimo Marteleto Nunes Rugani, Agnes Antônia Sampaio Pereira, Felipe Dutra Rêgo, Ana Cristina Vianna Mariano da Rocha Lima, Célia Maria Ferreira Gontijo, José Dilermando Andrade Filho

**Affiliations:** Grupo de Estudos em Leishmanioses–Centro de Pesquisas René Rachou–FIOCRUZ–MINAS–Avenida Augusto de Lima, 1715 Barro Preto, CP 1743, 30190–002 Belo Horizonte, MG, Brazil; Instituto Butantan, BRAZIL

## Abstract

Brazil is one of the most important endemic areas for leishmaniasis worldwide. Protected areas that are tourist attractions likely present an important risk of transmission of cutaneous leishmaniasis (CL). Furthermore, with the geographical expansion of visceral leishmaniasis (VL), several studies have recorded the occurrence of its vector, *Lutzomyia longipalpis*, and cases of human and canine VL in such tourist areas. The Parque Estadual do Sumidouro is an environmentally protected area located in the Brazilian Cerrado biome and in an important area endemic for leishmaniasis in the state of Minas Gerais. The purpose of this study was to monitor the sand fly fauna in areas of tourist activity in the park. Sampling was performed every month, from September 2011 to August 2013, using CDC light traps at six sites of differing environmental characteristics. Sampled specimens were identified following Galati (2003), and females were submitted to molecular techniques for the detection and identification of *Leishmania* DNA. A total of 4,675 sand fly specimens of 25 species belonging to nine genera were collected. The most abundant species were *Micropygomyia quinquefer*, *Lutzomyia renei* and *Pintomyia pessoai*, although only *Pi*. *pessoai* is implicated in the transmission of *Leishmania braziliensis*. The species accumulation curve reached saturation on the 16th sampling event. Species richness, diversity and evenness differed among the sampled areas. The seasonal curve was not determined by a single unique species, and no single species was the most abundant in all environments sampled. The main vector of *Leishmania* (*Leishmania*) *infantum*, *Lutzomyia longipalpis*, accounted for only 5.35% of the specimens collected. Proven or suspected vectors of *Leishmania* (*Viannia*) *braziliensis* were recorded, and one female of the *cortellezzii* complex tested positive for *Le*. *braziliensis* DNA. Even with a low infection rate (0.62%), these data indicate the circulation of the parasite and reinforce the need for entomological and epidemiological surveillance in the park and its surroundings.

## Introduction

Brazil is one of the most important areas of the world for the occurrence of endemic leishmaniasis. Given its high incidence and mortality rates in Brazil, much of the current attention has been given to urban foci of visceral leishmaniasis (VL). However, cutaneous leishmaniasis (CL) presents significant health problems in wild, rural and even urban and peri-urban areas [1] [2].

Environmentally protected areas that serve as tourist attractions may present an important CL transmission risk in southeastern Brazil. Typically, occupational exposure to CL transmission occurs when individuals are exposed to the focus of the disease, however, autochthonous cases have been recorded in urban and peri-urban areas [2]. With the geographical spread of VL, several studies have recorded populations of *Lu*. *longipalpis* and cases of human and canine VL in areas of more recent human settlement, including tourist areas [3] [4].

A survey of the protected area “Parque Estuadual do Alto Ribeira”, situated in a region endemic for CL, found the sand fly *Nyssomyia intermedia* (Lutz & Neiva, 1912), one of the main vectors of *Le*. *braziliensis*, to be the sixth most abundant sand fly species. This species had the highest rates of incidence in the camping area of the park where *Lu*. *longipalpis* was also recorded. Another study conducted in Bonito City, one of the main ecotourism destinations of Brazil, reported a high abundance of *Lu*. *longipalpis* as well as a high incidence of canine seroprevalence for *Leishmania*. Furthermore, *Nyssomyia whitmani* (Antunes & Coutinho, 1939), another main vector of *Le*. *braziliensis*, has been recorded in Bonito City. [5],[6].

The Parque Estadual do Sumidouro (Sumidouro State Park)–PES-, is situated in the Cerrado biome, known also as the Brazilian Savannah. The Cerrado is the second largest terrestrial biome in South America and occupies 22% of the land area of Brazil. Although the Cerrado is considered a global biodiversity hotspot, it is experiencing a remarkable amount of habitat destruction due to human occupation and activities such as urbanization and agricultural and coal production [7]. Such anthropogenic modifications can favor the occurrence of pathogens through the elimination of the natural habitats of reservoirs and vectors, thus causing the life cycles of these species to adapt to the modified environment [8], [9].

Knowledge of the risk of exposure to residents and tourists in these areas is essential for the surveillance and management of potential cases of leishmaniasis. Epidemiological data for areas frequently visited could be instrumental for the accurate diagnosis of leishmaniasis, and for the selection of treatment strategies, especially concerning tourists that return to non-endemic areas [10].

The purpose of the present study was to describe the sand fly fauna in PES, an area of protected Cerrado. The area is endemic for leishmaniasis, and has documented cases of both visceral and cutaneous human leishmaniasis. Located in PES is Lapinha’s cave, a taxonomically and biologically important site for the collection of *Lutzomyia longipalpis* [11] [12] [13] [14] [15].

## Materials and Methods

### Study area and collection of specimens

**Fig 1:**
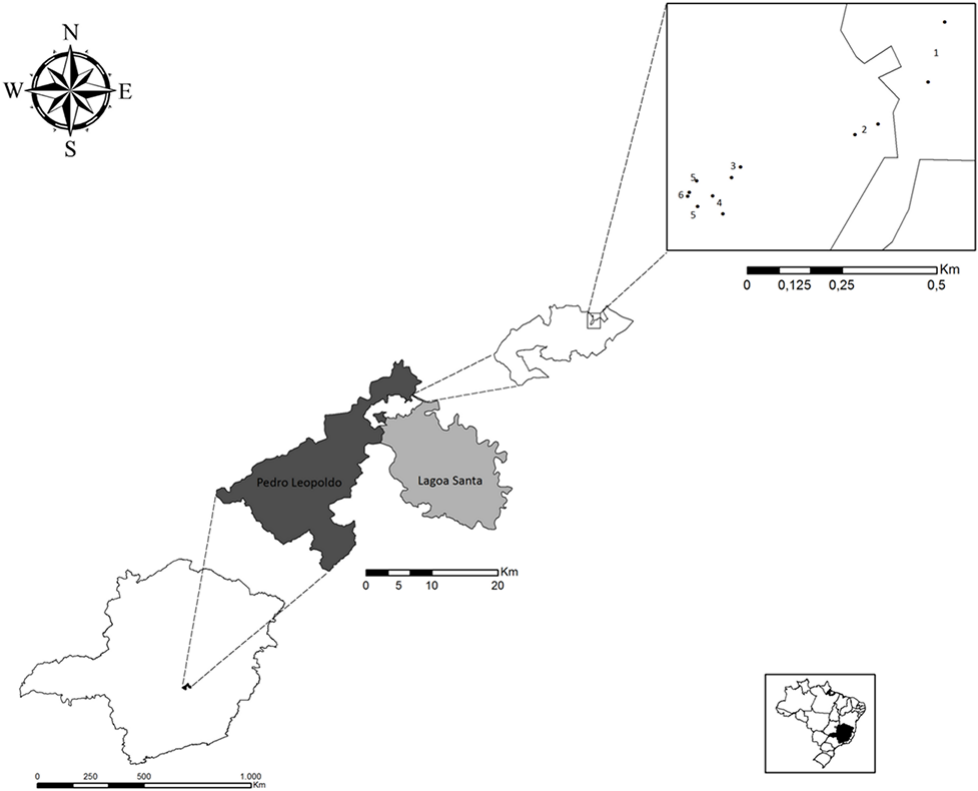
Map showing the location of PES and the location of sampling sites in the park. (1)—Park surroundings—peridomestic areas; (2)—Cerrado 1; (3)—Cerrado 2; (4)—Rupicolous vegetation; (5)—Cave entrance; (6)—Cave surroundings.

Sand flies were collected from six localities within PES of varying environmental characteristics, including type of vegetation, presence of rock formations, and degree of human disturbance. The PES is a thirteen-hundred hectare environmentally protected area located about 50 km from the city of Belo Horizonte, the capital of the state of Minas Gerais. The park is located in the Lagoa Santa karst region, with the relief being formed of carbonate rocks that are prone to dissolving with water [16] (Fig 1).

The environment of each sampling site was briefly described considering characteristics that may favor the occurrence of sand flies. Environmental characteristics considered were those that related to:

* Breeding, shelter and feeding on plants; presence and type of vegetation; presence of water collection sites; and the presence of rocks.
* Haematophagic habit: presence of local fauna, including dogs.

The environmental characteristics of the collection sites are briefly described in Table 1. The area sampled was 0.08 km^2^, and the traps were arranged in a non-linear transect of 1000 meters. The selection of collection sites followed two criteria: (1) the sampling of all of the different environments of the park considering vegetation, topography and anthropogenic modification; (2) proximity to the trail most commonly used by tourists (the primary reason for the entomological surveillance program).

**Table 1:** Environmental characteristics of the sand fly collection sites in PES from September 2011 to August 2013.

Collection site	Type of vegetation	Presence of livestock and dogs	Presence of stream, pond or dam	Presence of local fauna	Presence of rocks
**Park surroundings-–peridomestic areas**	Forested/semi-deciduous forest with high level of anthropic modification	Hen house and/or dog kennel	No	Yes	No
**Cerrado 1**	Forested/semi-deciduous forest	Wandering dogs	No	Yes	No
**Cerrado 2**	Wooded area/deciduous forest	Wandering dogs	No	Yes	No
**Rupiculous vegetation**	Wooded area/rupicolous vegetation	Wandering dogs	No	Yes	Yes
**Cave entrance**	-	No	Yes	Yes	Yes
**Cave surroundings**	Wooded area/rupicolous vegetation	Wandering dogs	No	Yes	Yes

Collection procedures were approved by the Ministério do Meio Ambiente do Brasil (Ministry of Environment of Brazil)—(SISBIO: 15,237 and 30297–2) and the Instituto Estadual de Florestas de Minas Gerais (State Forestry Institute of Minas Gerais) (072/11). Sampling took place over two years and was performed every month over three consecutive days using HP-model CDC light traps [17] arranged uniformly in the different study areas.

Twelve light traps were used: two traps were placed in the backyards of houses located in the park surroundings (peridomestic areas) and 10 traps were placed inside the park itself. Of the traps placed within the park, two were placed in forested/semi-deciduous forest (Cerrado 1), two in wooded/deciduous forest (Cerrado 2), two in rupicolous vegetation, two next to the entrance to the Lagoa do Sumidouro cave, and two in the area surrounding the cave.

The sampling transect was about 1000 meters in length (corresponding to a section of the main tourist trail of the park), with an average of 200 meters between trap sites. A total of only 0.08km^2^ (6.15%) of the entire 13,000 km^2^ of the park were sampled. Sampling took place from September 2011 to August 2013, with a total sampling effort of 20.736 hours.

Trapped specimens were identified according to the classification proposed by Galati (2003). Females belonging to the species *Evandromyia sallesi* (Galvão & Coutinho, 1940) and *Evandromyia cortelezzii* (Brethes, 1923), and belonging to the species *Pintomyia mamedei* (Oliveira, Afonso, Dias & Brazil, 1994) and *Pintomyia christenseni* (Young & Duncan, 1994), were identified as belonging to the *cortelezzii* complex and the *mamedei* complex, respectively, when morphological differentiation was inconclusive.

Female specimens collected up to April 2013 were processed for the detection of *Leishmania* DNA. These specimens were stored in DMSO 6% and frozen at -20°C. At the time of identification, the last three abdominal segments and the head of each female specimen were removed and mounted in Berlese liquid. Simultaneously, the gut was checked for the presence of blood. The remaining specimens were placed in 70% alcohol, prepared and mounted in Canada Balsam.

### DNA extraction and polymerase chain reaction

DNA extraction employed the Puregene QIAGEN Gentra kit. The molecular target chosen for *Leishmania* DNA detection was the Internal Transcribed Spacer I (ITS I) of rDNA. [18]. The reaction protocol used was that established by Schonian et al 2003 [18] with some modifications. The positive controls were DNA of four reference strains: *Leishmania braziliensis* (MHOM/BR/75/M2903), *Leishmania guyanensis* (MHOM/BR/75/M4147), *Leishmania infantum* (MHOM/BR/74/PP75), and *Leishamania amazonensis* (IFLA/BR/67/PH8) at a concentration of 20 ng/μl. For species identification the amplified products were digested by the enzyme Hae III as described by Schonian et al 2003 [18].

If the restriction fragment analysis did not produce visible DNA bands, the whole fragment resulting from the PCR amplification of the ITS-1 region was sequenced. To purify the product QIAquick PCR Purification Kit (Qiagen) was used following the manufacturer’s instructions. Next, a mixture was prepared containing 1μL of purified products, 1μL of each primer at a concentration of 5 pmol, antisense or sense (in separate tubes), 1μL 5x Sequencing Buffer, 1μL of BigDye Terminator v3.1 Cycle Sequencing, and distilled water for a final volume of 10 μL. The program used 35 cycles alternating between 95°C for 15 seconds and 65°C for 15 seconds. The ITS-1 fragment sequences were read using an ABI 3730xl DNA Analyzer automatic DNA sequencer.

### Data analysis

The focus of the present study was to perform a descriptive analysis of sand-fly population patterns in areas of Cerrado with different kinds of vegetation. Margalef diversity and J evenness indices were used for ecological analyses of the study areas since they are non-parametric indices, which are less influenced by differing sampling efforts. The Margalef diversity index is used to summarize information about the number of species and their abundance, and is employed to compare samples and locations. The J evenness index is used to describe the distribution of collected specimens among the species present, and thus is used to make inferences about species dominance in a population [19].

Descriptive analyses of all data were performed using Microsoft Excel (Office 2003). Graph Pad Prisma 4.0 software was used for statistical analyses and the PAST program was used for the ecological analyses. The entomological sampling sites were georeferenced using GPS—GARMIN eTrex-H for the spatial analysis of vector species (suspected and confirmed) population density. The coordinates and presence/absence data of vectors for each site were analyzed using ArcGIS 9.3 software.

For each study site, kernel estimation was used to infer vector density. This is a non-parametric statistical method of interpolation that identifies sites with the highest occurrence of a given event [20]. For kernel estimation in the present study, sand fly species were categorized as vectors (suspected or confirmed) or non-vectors of *Leishmania* species.

Sequences of the ITS-1 fragment were analyzed using Finch TV software (Geospiza, Inc.). Alignment of the sequences with sequences obtained from GenBank was performed using BLAST (www.ncbi.nlm.nih.gov/BLAST).

## Results

### Sand fly fauna and environmental characteristics

**Fig 2:**
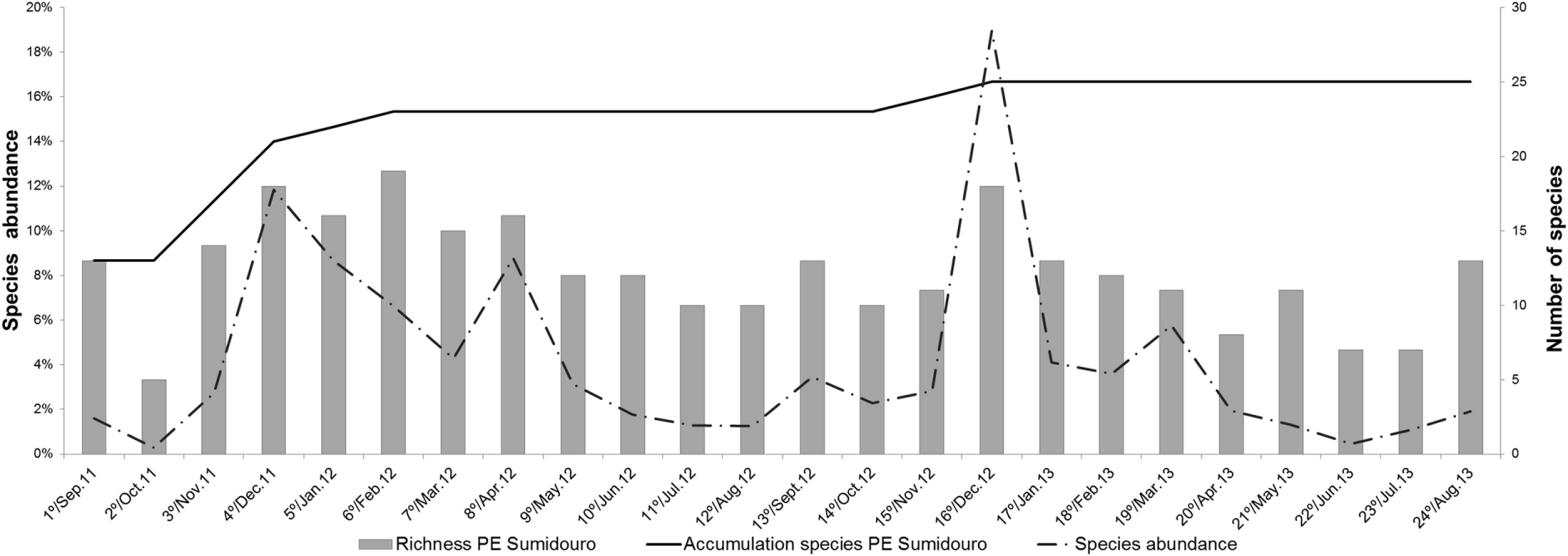
Species accumulation curve, abundance, and species richness of the sand fly fauna of PES from September 2011 to August 2013.

A total of 4,675 sand fly specimens belonging to nine genera and 25 species were collected. The species with the highest abundance was *Micropygomyia quinquefer* (Dyar, 1929), which accounted for 37.54% of the total. The next most abundant species were *Lutzomyia renei* (Martins, Falcão & Silva, 1957) and *Pintomyia pessoai* (Barretto & Coutinho, 1940), with 22.67% and 9.52%, respectively (Table 2). Considering these three most abundant species, only *Pi*. *pessoai* is implicated in the transmission of *Leishmania braziliensis*. Species richness and diversity were relative high among the samplings (Tables 2 and 3, Fig 2). The species accumulation curve reached saturation at the 16th sampling event when the value stabilized at 25 species. The six sites had distinctly different values for the species richness, diversity, abundance, and equitability indices. Even in the small region of this protected area sampled, there is considerable heterogeneity in the sand fly composition among sampled sites (Tables 1 and 2).

**Table 2:** Species of sand flies collected by site in the PES, sex, and total percentage from September 2011 to August 2013.

Sand flie especies	Collected specimens per species		Number of collected specimens per study environments						Number of collected specimens per sex	
	Total number of specimens	% of collected specimens per species	Park surroundings	Cerrado 1	Cerrado 2	Rupicolous	Cave entrance	Cave surroundings	♂	♀
*Brumptomyia brumpti*	48	1.03	0	33	12	0	0	3	29	19
*Brumptomyia pintoi*	14	0.30	0	6	7	0	1	0	8	6
Brumptomyia sp.	24	0.51	0	10	4	1	3	6	-	24
*mamedei* complex	48	1.03	1	22	11	10	1	3	-	48
*cortelezzii* complex	168	3.59	57	5	57	11	23	15	-	168
*Evandromyia bacula*	3	0.06	0	1	0	1	0	1	-	3
*Evandromyia cortelezzii*	137	2.93	62	1	25	22	17	10	122	15
*Evandromyia lenti*	22	0.47	5	2	12	2	0	1	10	12
*Evandromyia sallesi*	69	1.48	24	2	24	14	2	3	3	66
*Evandromyia spelunca*	3	0.06	2	0	0	0	1	0	-	3
*Evandromyia termithophila*	18	0.39	2	6	10	0	0	0	6	12
*Evandromyia* sp.	1	0.02	0	0	0	0	1	0	1	-
*Lutzomyia longipalpis*	250	5.35	1	3	20	57	152	17	134	116
*Lutzomyia renei*	1060	22.67	1	5	4	18	1021	11	574	486
*Lutzomyia* sp.	65	1.39	4	14	8	14	20	5	40	25
*Micropygomyia quinquefer*	1755	37.54	2	1	50	1258	359	85	687	1068
*Migonemyia migonei*	47	1.01	11	0	8	4	19	5	14	33
*Nyssomyia intermedia*	57	1.22	7	2	4	9	11	24	16	41
*Nyssomyia neivai*	3	0.06	0	1	0	1	1	0	2	1
*Nyssomyia* sp.	1	0.02	0	1	0	0	0	0	-	1
*Nyssomyia whitmani*	73	1.56	3	11	8	7	23	21	11	62
*Pintomyia christenseni*	22	0.47	0	14	5	3	0	0	11	11
*Pintomyia fischeri*	11	0.24	0	4	2	4	0	1	5	6
*Pintomyia mamedei*	4	0.09	0	3	0	1	0	0	-	4
*Pintomyia monticola*	6	0.13	0	1	2	2	0	1	1	5
*Pintomyia pessoai*	445	9.52	4	227	106	63	4	41	294	151
*Pintomyia* sp.	2	0.04	0	1	1	0	0	0	1	1
*Psathyromyia aragaoi*	74	1.58	0	67	5	1	0	1	39	35
*Psathyromyia aragaoi* c.f.	3	0.06	0	3	0	0	0	0	-	3
*Psathyromyia barretoi barretoi*	5	0.11	0	5	0	0	0	0	-	5
*Psathyromyia brasiliensis*	32	0.68	0	22	10	0	0	0	13	19
*Psathyromyia lutziana*	129	2.76	5	93	17	5	5	4	51	78
*Psathyromyia* sp.	1	0.02	0	1	0	0	0	0	-	1
*Sciopemyia sordellii*	75	1.60	1	8	14	3	24	25	18	57
**Totals**	4675	100	192 (4.11%)	575 (12.30%)	426 (9.11%)	1511 (32.32%)	1688 (36.11%)	283 (6.05%)	2090	2585

**Table 3:** Diversity indices for the sand fly fauna of the collection sites in PES from September 2011 to August 2012.

Indices	Sites					
	Park surroundings	Cerrado 1	Cerrado 2	Rupicolous	Cave entrance	Cave surroundings
**Margalef diversity index SP Sumidouro**	2.671	3.52	3.251	2.467	1.756	2.889
**Equitability_J index SP Sumidouro**	0.6749	0.6068	0.8145	0.2471	0.4382	0.7478

The area referred to as Cerrado 1 (forested/semi-deciduous forest) had the highest values for the diversity index, followed by Cerrado 2 (wooded area/deciduous forest); both areas had high values of equitability. The localities with the lowest values for species richness were the Cave entrance and the Rupicolous area; both had the lowest values of equitability as well. These latter two environments, however, had the highest values for relative abundance (Table 2).

There is no single species that dominates all of the studied environments in the park. For instance, in the park surroundings, specimens of the *cortelezzii* complex and *Evandromyia cortelezzii* were the most predominantly collected, whereas in areas of Cerrado 1 (forested/semi-deciduous forest) and Cerrado 2 (wooded areas/deciduous forest) *Pi*. *pessoai* predominated. The most abundant species in the area of rupicolous vegetation and in the environment surrounding the cave was *Mi*. *quinquerfer*, and *Lu*. *renei* was the most common species in the cave entrance. The vector species *Lu*. *longipalpis*, *Ny*. *intermedia* and *Ny*. *whitmani*, were collected from all environments studied in the park. *Migonemyia migonei* (France, 1920) and *Pintomyia fischeri* (Pinto, 1926) were collected from five and four study areas, respectively.

**Fig 3:**
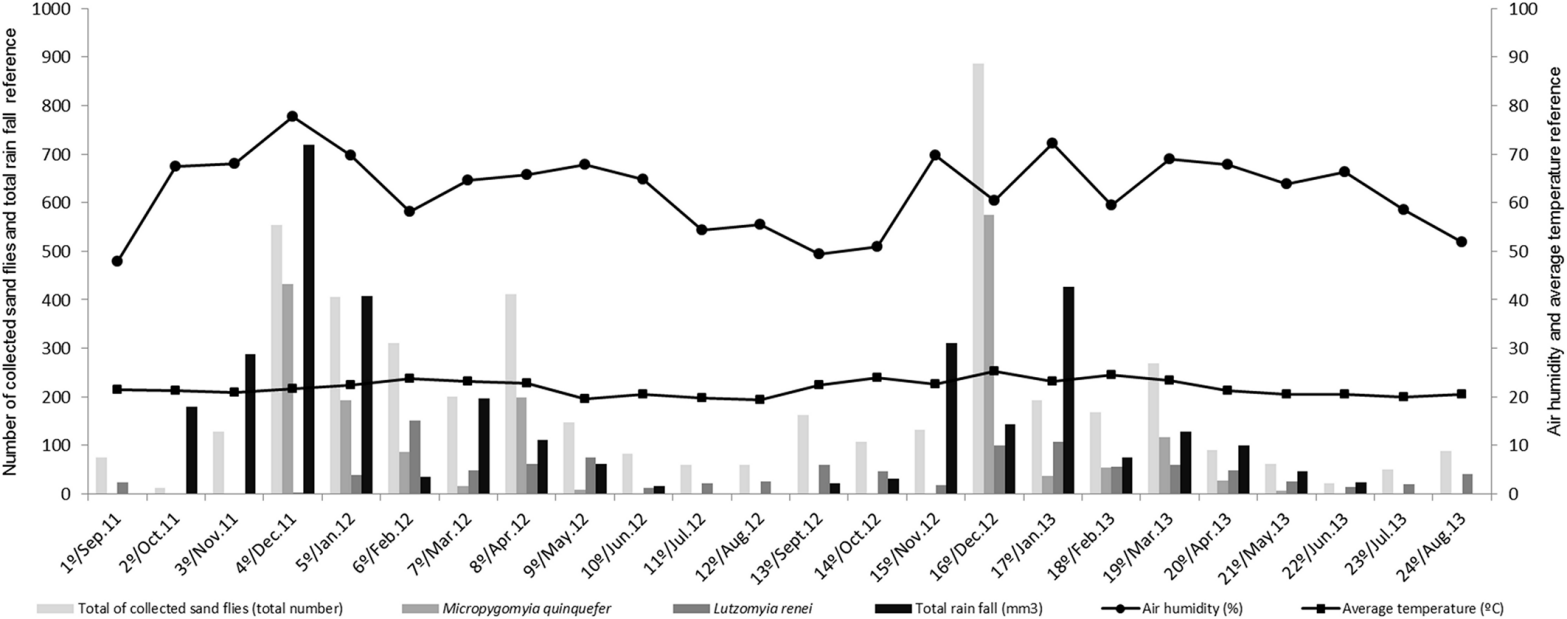
Relationship between seasonal variation of sand fly abundance and the climate parameters of total precipitation, relative humidity and mean temperature in PES from September 2011 to July 2013. The left Y-axis references the number of sandflies collected (total number) and the precipitation rate (mm^3^)—these data are represented by bars. The right Y-axis references the average temperature (°C) and relative air humidity (%)—these data are represented by lines with markers.

During both years of sampling the most productive period of sand fly collection was from December to April, whereas the least productive was in June and July (Figs 2 and 3). The species with the highest abundance values, *Mi*. *quinquefer* and *Lu*. *renei*, did not determine the pattern of the seasonal curve (Fig 3). The analyses of the seasonal curve in relation to the climatic parameters showed peaks of sand fly abundance during warm and humid periods. Among the climatic parameters analyzed, only relative humidity was not statistically correlated with the seasonal curve [Temperature (p-value < 0,0001 – Spearman correlation test), Precipitation (p-value = 0,0013 – Spearman correlation test), Air relative humidity (p-value = 0,0984 – Spearman correlation test)].

### *Leishmania* DNA detection and identification

Of the total of 2,380 female specimens analyzed for the detection of *Leishmania* DNA, 1,014 were processed individually and 1,366 were grouped into 153 pools. These female sand flies were grouped according to species, collection date and collection site, with 2 to 20 individual sand flies in each pool (mean = 14). Only four individually processed samples tested positive for ITS-1 PCR (Table 4). The results of the RFLP reactions were not informative due to low DNA concentrations, and so the identification of samples was performed through the sequencing reaction. Only one sample was identified as *Le*. *braziliensis* and it was from a female of the *cortelezzii* complex. Two samples were identified as *Crithidia fasciculata*, one as a female *Ev*. *sallesi* and another as a female *Psathyromyia lutziana* (Costa Lima 1932). A female *Sciopemyia sordellii* (Shannon & Del Ponte, 1927) had a positive reaction for trypanosomatids. The natural infection rate of females of the *cortelezzii* complex by *Le*. *braziliensis* was 0.62%.

**Table 4:** Results of DNA detection and identification of species of Trypanosomatidae according to species of sand fly, collection sites, and collection date in PES from September 2011 to August 2013.

Species	Sites	Colection date	ITS1-PCR	RFLP profile	Sequencing
*Ev*. *sallesi*	Park surroundings—peridomestic areas	Mar/12	Positive	Undefined	*Crithidia fasciculata* (id: 100%, ref: HM004585.1)
*Pa*. *lutziana*	Park surroundings—peridomestic areas	Mar/12	Positive	Undefined	*Crithidia fasciculata* (id: 100%, ref: HM004585.1)
*Sc*. *sordellii*	Cave surroundings	Mar/12	Positive	Undefined	Trypanosomatidae (id: 93%, ref: JN673399.1)
*cortelezzii* complex	Cerrado 2	Dec/12	Positive	Undefined	*Leishmania braziliensis* (id: 100%, ref: JX448549.1)

### Vectors density analyses

**Fig 4:**
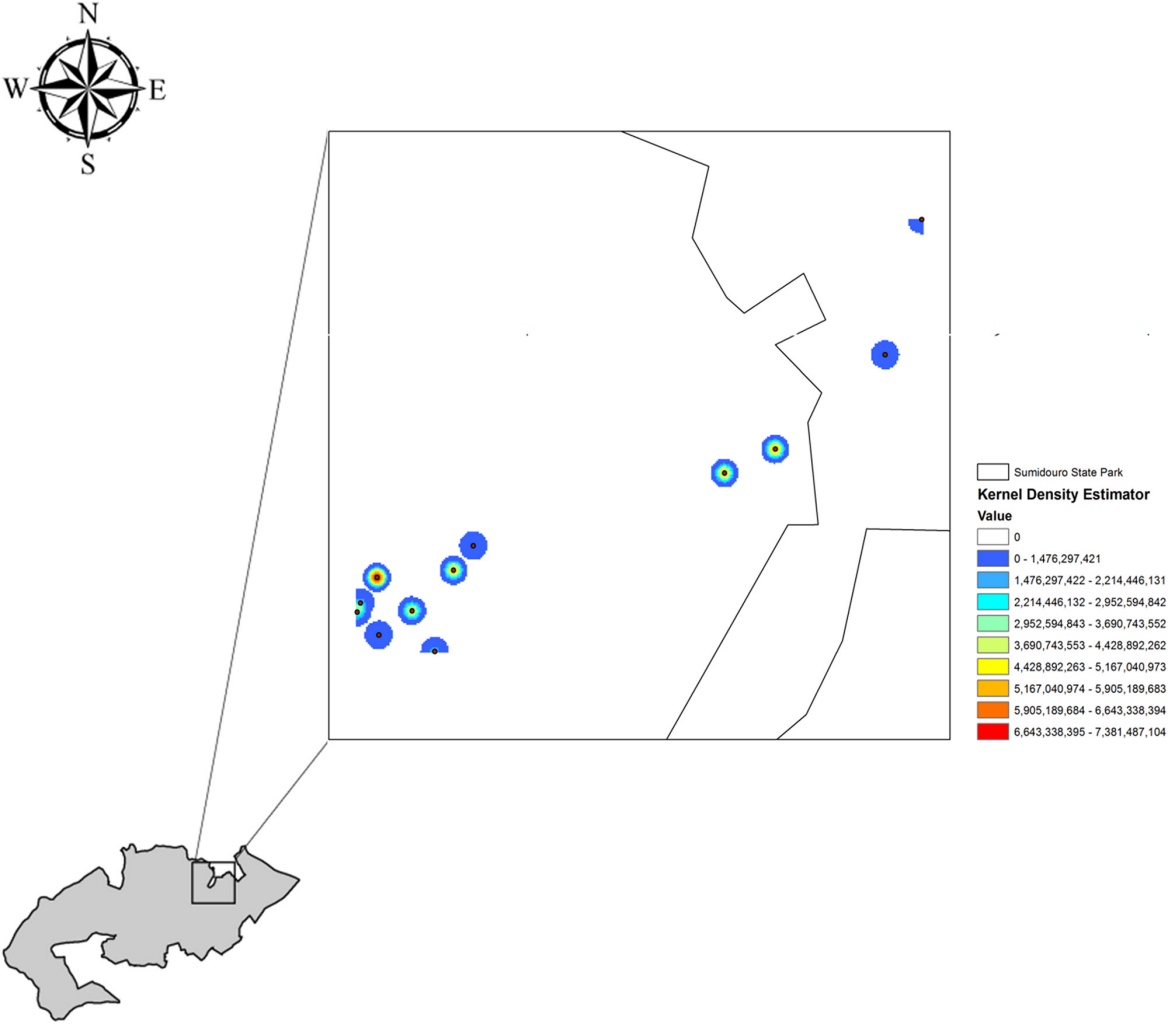
Map showing the Kernel density analysis of vectors in PES.

The map of suspected or proven vector species shows that they were distributed among all of the collection sites in PES, with the highest concentration of vector species occurring at the cave entrance and the areas of forested/semi-deciduous forest and wooded/deciduous forest. The area of rupicolous vegetation had intermediate vector densities, whereas the peridomestic localities surrounding the park had the lowest vector densities (Fig 4).

## Discussion

The PES is located in the municipalities of Lagoa Santa and Pedro Leopoldo, both of which have historical records of human cases of VL and CL and report cases of canine leishmaniasis. From 2009 to 2013, three cases of VL were documented in Lagoa Santa and two in Pedro Leopoldo. During the same time period, three cases of CL were reported in Lagoa Santa and six in Pedro Leopoldo [21].

Among the proven or suspected vector species collected in the park, *Lu*. *longipalpis* accounted for 5.35%. Although this species was collected in all of the environments sampled, it was most abundant at the cave entrance. This species was also collected in high numbers in less anthropogenetically influenced habitats, with only one specimen being collected in the peridomestic environment.

The data of this study are consistent with the hypothesis that different populations of *Lu*. *longipalpis* have different behavior patterns in different environments. However, it may be that this species merely possesses an enormous ability to adapt to anthropogenically modified environments [22], [23], [24], [25].

A large number of species that are suspected or proven vectors of *Le*. *braziliensis* were documented in PES, such as *Ny*. *whitmani* [26]. Moreover, *Le*. *infantum* DNA was detected in females of *Ny*.*whitmani* in different areas of Minas Gerais State [27], [28]. This species has a tendency to adapt to anthropogenically influenced areas in spite of its greatest abundances in natural areas and forest environments [29], [30], [31].

Specimens of *Ev*. *sallesi* and *Ev*. *cortelezzii*, as well as specimens of the *cortelezzii* complex, are suspected of participating in the transmission cycles of *Leishmania* sp. These species are frequently collected in peridomestic environments, and the DNA of *Le*. *infantum* has been detected in both *Ev*. *sallesi* and *Ev*. *cortelezzii*. Furthermore, specimens of the *cortelezzii* complex have been found positive for *Le*. *braziliensis* DNA [28], [32]. In southeastern Brazil, *Ny*. *intermedia*, *Nyssomyia neivai* (Pinto, 1926), *Mi*. *migonei*, *Pi*. *pessoai* and *Pi*. *fischeri* have been incriminated in the transmission of *Le*. *braziliensis*, [28], [29], [33], [34], [35], [36], [37], [38].

Species whose participation in the transmission cycles of leishmaniasis need to be further clarified were also collected, such as *Evandromyia lenti* (Mangabeira 1938) and *Sc*. *sordellii* [27], [39], [40], [41]. The remaining species of sand flies recorded in PES are not suspected to be involved in the transmission cycles of *Leishmania* spp.

So far the indices of species richness, diversity and abundance recorded among the park localities have been described through simple descriptive analysis. Additional analyses using non-parametric statistical methods provide a similar interpretation [19], [42].

In every sampling, the study areas that had elevated values of diversity remained relatively stable throughout the study period, which agrees with what is expected of an environmentally preserved area (Tables 1 and 2). In PES evenness was high and there was not a single species that represented more than 40% of the sand fly specimens collected in the park (Table 1). These data agree with other studies in which natural areas tended to have greater species richness and diversity. For example, a study conducted on Ilha Grande, RJ, Brazil, a recent transmission area of *Le*. *infantum*, higher species richness and abundance were found in areas with lower anthropogenic modification [38].

In a study in the state of Espírito Santo, Brazil, a similar pattern was found in tropical forest areas and the authors emphasize that conserved areas have greater opportunities for shelter and more sources of food for sand flies [43]. These data concur with classical ecological theories that postulate that the greater diversity of habitats favors the greater biodiversity of species [44].

The present data analysis shows that the environmental diversity of the park holds highly differentiated faunistic compositions even in proximate sampling sites. For instance, the species of the genus *Brumptomyia* [(*Brumptomyia brumpt* (Larrousse, 1920) and *Brumptomyia avellari* (Costa Lima, 1932)] were not recorded from the peridomestic environments and had higher relative abundances in Cerrado 1 and Cerrado 2. This pattern of occurrence is consistent with other faunal surveys [31], [45], [46]. Similar distribution patterns were also observed for other species (Table 1).

Although sand flies have not traditionally been considered in ecological conservation studies, the data herein corroborates and reinforces studies that call for better strategies to protect the Brazilian Cerrado biome [47]. Given the ecological complexity of the sand fly fauna of PES, it could be inferred that other invertebrate groups of the region, that have not yet been investigated, could be under risk due to impacts to the entire biome. These findings in PES agree with general descriptions of the Cerrado biome; the environmental diversity of Brazilian Cerrado forms a complex mosaic of habitats with different vegetation types, which contributes to a non-uniform species distribution [7].

The biodiversity of the park area is clearly demonstrated by the seasonal curve, which is influenced by several species. For example, *My*. *quinquerfer* and *Lu*. *renei* are responsible for the highest peaks of sand fly occurrence in the park, however, their individual patterns differ. The pattern of the seasonal curve in PES is in accordance with other studies carried out in the city of Belo Horizonte and in the region of Lapinha’s cave [11], [30]. However, these results differ from others studies conducted in Belo Horizonte in which peaks in warmer and rainy months are not so evident [48]. These results demonstrate that entomological monitoring is required for leishmaniasis prevention and control measures based on vector control strategies because seasonal patterns of variation are prone to change due to environmental factors and insect community composition.

Only one female of the *cortelezzi* complex was found to be positive for *Le*. *braziliensis* DNA. Even if representing a low infection rate (0.62%), this observation indicates the circulation of the parasite in the area and reinforces the need for entomological and epidemiological surveillance. It is important to note that the molecular marker used in this study, the ITS-1 region, was specifically designated for detecting the genus *Leishmania* [49], however, in this work the region was able to detect another genus of the family Tripanosomatidae; two females tested positive for the genus *Chritidia*.

The vector distribution map summarizes the distribution of epidemiological risk since it shows significant vector density. As reported in other studies, the risk is not equally distributed among sampling localities; there is a higher record of vector species, or potential vectors, in locations with certain environmental characteristics [30], [50], [51]. Modern public health practices now possess the tools necessary to analyze key points in the relationship between the events involved in human diseases [52], [53].

These results underscore the need for entomological vigilance in PES where the presence of vectors, and the proximity of important endemic areas, suggest the possible establishment of cycles involving the parasites. A total of 14 sand fly species with proven or suspected involvement in the transmission cycles of *Le*. *infantum* and *Le*. *braziliensis* in Brazil were recorded in PES. The main vector species of *Le*. *infantum*, *Lu*. *longipalpis*, was detected in this study, whereas 13 species related to the transmission cycles of *Le*. *braziliensis* were recorded. Furthermore, the tourist potential of the park and, the tendency for an increasing number of visitors, reinforce the need to establish entomological monitoring in the area. This type of situation has been described in other tourist areas in Brazil [38], [54].

It is not possible to ignore human factors in considering control measures of endemic diseases and improvements are needed in the way of entomological monitoring in order to employ preventive actions. This need is reinforced by the increasing amount of tourism in the area [55].

## Conclusions

Our data show the presence of confirmed and suspected vector species of *Leishmania infatum* and *Leishmania braziliensis* at all sampling points along the main tourist trail in PES. This, along with the finding of *Le*. *braziliensis* in one female of the *cortellezzi* complex, demonstrate the need for continuing entomological and epidemiological surveillance in PES.
